# African American Unemployment and the Disparity in Periviable Births

**DOI:** 10.1007/s40615-021-01022-7

**Published:** 2021-03-30

**Authors:** Ralph Catalano, Deborah Karasek, Tim Bruckner, Joan A. Casey, Katherine Saxton, Collette N. Ncube, Gary M. Shaw, Holly Elser, Alison Gemmill

**Affiliations:** 1 School of Public Health, University of California, Berkeley, Berkeley, CA 94720 USA; 2 School of Medicine, University of California, San Francisco, San Francisco, CA 94143 USA; 3 Program in Public Health, University of California, Irvine, Irvine, CA 92697 USA; 4 Mailman School of Public Health, Columbia University, 722 West 168th Street, New York, NY 10032 USA; 5 Program in Public Health, Santa Clara University, 500 El Camino Real, Santa Clara, CA 95053 USA; 6 Department of Health Sciences, Northeastern University, 360 Huntington Ave., Boston, MA 02115 USA; 7 School of Medicine, Stanford University, Stanford, CA 94305 USA; 8 Population, Family and Reproductive Health, Johns Hopkins University, 615 N. Wolfe St., Baltimore, MD 21205 USA

**Keywords:** African American, Disparities, Periviable birth, Unemployment

## Abstract

Periviable infants (i.e., born before 26 complete weeks of gestation) represent fewer than .5% of births in the US but account for 40% of infant mortality and 20% of billed hospital obstetric costs. African American women contribute about 14% of live births in the US, but these include nearly a third of the country’s periviable births. Consistent with theory and with periviable births among other race/ethnicity groups, males predominate among African American periviable births in stressed populations. We test the hypothesis that the disparity in periviable male births among African American and non-Hispanic white populations responds to the African American unemployment rate because that indicator not only traces, but also contributes to, the prevalence of stress in the population. We use time-series methods that control for autocorrelation including secular trends, seasonality, and the tendency to remain elevated or depressed after high or low values. The racial disparity in male periviable birth increases by 4.45% for each percentage point increase in the unemployment rate of African Americans above its expected value. We infer that unemployment—a population stressor over which our institutions exercise considerable control—affects the disparity between African American and non-Hispanic white periviable births in the US.

## Introduction

Infants born before 26 complete weeks of gestation, who represent fewer than .5% of births in the US, account for approximately 40% of the nation’s infant mortality [1]. These “periviable” infants also account for nearly 20% of birth-related hospitalization costs [2]. African American women contribute about 14% of live births in the US, but these infants include nearly a third of all those born periviable [3]. As with all preterm births, males comprise the majority of periviable infants regardless of race and ethnicity [4].

We know more about the sequelae of periviable birth than we do of its causes [5]. Indeed, the National Institute of Child Health and Human Development has called for “exploratory and novel” research into periviable birth [6]. One novel approach views the majority of periviable births as post 20th week spontaneous abortions averted through medical intervention [7, 8]. Consistent with this view, late spontaneous abortions and periviable births share many characteristics including that small for gestational age males predominate among them [9–13]. Viewing periviable births as late spontaneous abortions averted through medical intervention implies that explanations of the latter may apply to the former [14]. At least half, and likely many more, of human conceptions end without elective abortion or live birth [15, 16]. This loss appears, early in gestation, disproportionately among female fetuses with chromosomal and genetic abnormalities [15]. After the first trimester, however, small for gestational age, but otherwise “normal,” males predominate among spontaneous abortions as they do among periviable births [9, 11, 17]. Much literature attributes this selection *in utero* against small males to mechanisms, conserved by natural selection, that historically averted maternal investment in offspring with low likelihood of surviving in environments threatening to frail infants particularly small males [14]. Indeed, small for gestational age and male sex remain among the strongest predictors of which infants die in stressful environments [17–19].

The epidemiologic literature that describes biological correlates of preterm birth, reports that “stressed” women appear at elevated risk [20]. The work argues that hormonal shifts associated with the human stress response likely accelerate the “pregnancy clock” [21]. Other work grounds this argument in evolutionary theory by suggesting that mechanisms conserved to realize the maternal fitness benefits of selection *in utero* include the stress response [22].

Consistent with stress-induced selection *in utero*, much literature attributes the disproportionate burden of spontaneous abortion among African American women to unequal distribution of toxic stressors and coping resources [23–29]. This literature reports that socially constructed racial/ethnic hierarchies not only induce these distributions but also directly stress people of color [30, 31]. Spontaneous abortion resulting from selection *in utero* would, therefore, exhibit patterns that reflect racial/ethnic hierarchies.

The circumstances summarized above lead to the argument that the racial disparity in male periviable births increases with the dose of any stressor suffered disproportionately by African Americans. We test that argument using the African American unemployment rate as an indicator of stress in the population. We chose the unemployment rate for several reasons. First, a rising rate implies a contracting labor market that stresses not just workers, but their families and social networks as well [32–34]. Rising unemployment also signals decreased coping resources, including income, among the unemployed and underemployed [35]. It also engenders anxiety via fear of job loss in the remainder of the labor force [36]. The unemployment rate, therefore, serves not only as a tracer of, but also a contributor to, the prevalence of stress in the population [37].

Second, African American workers appear especially vulnerable to contracting labor markets in part because a race-based hierarchy of employment opportunities [37] assigns them disproportionately to temporary, low-wage, jobs that remain the ‘last hired, first fired’ during economic downturns [38]. During the Great Recession, for example, African Americans lost more jobs, had a steeper reduction in income, and were more likely to lose health insurance than did non-Hispanic whites [39–42].

Third, the literature includes reports that contracting labor markets increase disparities in preterm birth [43] and in small for gestational age births [44] between African American and non-Hispanic white women. And last, we can do something about the disparity because public policy affects the incidence of unemployment.

## Methods

### Data

We constructed our dependent variable from anonymized data publicly available from the Centers for Disease Control and Prevention [45]. These data allowed us to calculate the monthly odds of a periviable birth (i.e., 20 0/7 through 25 6/7 weeks of gestation) among survivors to birth from monthly conception cohorts of males and females conceived by African American and non-Hispanic white women. We chose to analyze conception rather than birth cohorts because estimating the population at risk of periviable birth in the latter remains difficult. Most infants born in any month have been in gestation for more than 26 weeks making total births a highly inaccurate estimate of the population at risk of periviable birth. We reduce the error by assigning births to conception months based on gestational age at birth and by computing the odds of periviable to later births for each monthly conception cohort.

We derived our independent variable from data collected as part of the US Current Population Survey and published by the US Bureau of Labor Statistics [46]. More specifically, we used series LNS14000006 labeled as “Unemployment Rate: Black or African American, Percent, Monthly, Seasonally Adjusted.” An unemployed person has no paid job but has tried to find one in the last month. The unemployment rate is the percentage of the labor force (i.e., the sum of employed and unemployed persons) unemployed at the time of the survey.

We used data ranging from January 1998 through December 2016 (last month of available data at the time of our analyses). These 228 months included those with low, high, and historically typical values of both African American unemployment, as well as odds of periviable birth by sex and race/ethnicity. Live births over our test period included 5,596,032 African American and 21,397,800 non-Hispanic white males, as well as 5,418,648 African American and 20,305,452 non-Hispanic white females.

### Analyses

The argument that societally controlled stressors on African Americans increase the racial disparity in periviable births predicts that the odds ratio of such births will rise above statistically expected values when the unemployment rate in African American communities rises above its expected values. Our test, therefore, requires that we arrive at statistically expected values of both variables. Tests of association typically assume normal and independent distribution of variables. These assumptions allow specifying the mean as the expected value. Variables measured over time, however, often violate these assumptions by exhibiting “autocorrelation” in the form of secular trends, cycles, or the tendency to remain elevated or depressed, or to oscillate, after high or low values. The expected value of an autocorrelated series is not its mean but rather the value extrapolated from autocorrelation. Following practice dating at least to Fisher [47], and adapted by epidemiologists [48], we solved this problem by identifying the time-series model that best fits observed autocorrelation in both our variables. We used the most developed and widely disseminated type of such modeling. The method, devised by Box and Jenkins [49], identifies which of a very large family of models best fits measurements made serially in time or space. The Box and Jenkins approach attributes autocorrelation to integration, as well as to "autoregressive" and "moving average" parameters. Integration describes secular trends and strong seasonality. Autoregressive parameters best describe patterns that persist for relatively long periods, while moving average parameters parsimoniously describe less persistent patterns.

Our test proceeded through the following steps.

1. We calculated the sex-specific odds ratio of periviable birth among survivors to birth from cohorts conceived by African American and non-Hispanic white women in the 228 months beginning January 1998 and ending December 2016. We transformed the odds ratio to its natural logarithm to normalize its distribution and to allow interpretation of results as percentage change in the odds ratio associated with a 1-unit change in the independent variable.
2. We used the Box–Jenkins methods to detect and model autocorrelation in the sex-specific odds ratios calculated in step 1. The fitted values of these models became the “counterfactuals” for our test implying that the model error terms measure the difference between the observed odds ratios and those expected from autocorrelation.
3. We used the Box–Jenkins methods to detect and model autocorrelation in the African American unemployment rate. The model error terms measure the difference between the observed and expected (i.e., from autocorrelation) unemployment rate and serve as our independent variable.
4. We estimated a test equation formed by adding the error terms from the model for unemployment estimated in step 3 to the odds ratio model estimated in step 2. We specified the unemployment error terms in the same month as conception (i.e., both in month *t*), as well as in month *t+1* to *t+6*. We stop at *t+6* because all member of the cohort conceived in month *t* but exposed to unemployment at *t+7* would have completed 28 weeks of gestation (i.e., beyond risk of periviable birth). The full test equation, which we estimated separately for males and females, was as follows.

$$ \left({\mathrm{p}\mathrm{ba}}_{\mathrm{t}}/{\mathrm{oba}}_{\mathrm{t}}\right)/{\left({\mathrm{p}\mathrm{bw}}_{\mathrm{t}}/{\mathrm{obw}}_{\mathrm{t}}\right)}^{\mathrm{e}}=\mathrm{C}+{\upomega}_0{\mathrm{X}}_{\mathrm{t}+}{\upomega}_1{\mathrm{X}}_{\mathrm{t}+1}+\dots {\upomega}_6{\mathrm{X}}_{\mathrm{t}+6}+\left(1-{\uptheta \mathrm{B}}^{\mathrm{q}}\right)/\left(1-{\upvarphi \mathrm{B}}^{\mathrm{p}}\right){\upalpha}_{\mathrm{t}} $$

pba_t_ is the number of periviable African American births yielded by the cohort conceived in month t. oba_t_ is the number of other live African American births yielded by the cohort conceived in month t. pbw_t_ is the number of periviable non-Hispanic white births yielded by the cohort conceived in month t. obw_t_ is the number of other live non-Hispanic white births yielded by the cohort conceived in month t. C is a constant. X_t_ to X_t+6_ are time series of error terms from the Box–Jenkins model estimated in step 3 for the African American unemployment rate in months *t* to *t+6*. ω_0_ through ω_6_ are estimates of association. θ is a Box–Jenkins moving average parameter. φ is a Box–Jenkins autoregressive parameter. B^p^ and B^q^ are “backshift operators” or the value of (pba_t_/oba_t_)/(pbw_t_/obw_t_)^e^ at time t-p or t-q. α_t_ is the residual of the model at month *t*. The methods applied in step 2 determine whether the test equation includes either, or both, autoregressive (i.e., φB^p^) or moving average (i.e., θB^q^) parameters, as well as the values of p and q.

The argument that exogenous stressors on the African American population increase racial disparities in periviable births among males past the first trimester of gestation predicts that one or more of the coefficients for unemployment 3 through 6 months after conception will detectably exceed 0. Consistent with recommendations in the literature concerned with replication of results [50, 51], we set our criterion for detection at *P* < 0.01 (single-tailed test) and provide standard errors to allow readers to set other criteria.

## Results

**Fig. 1:**
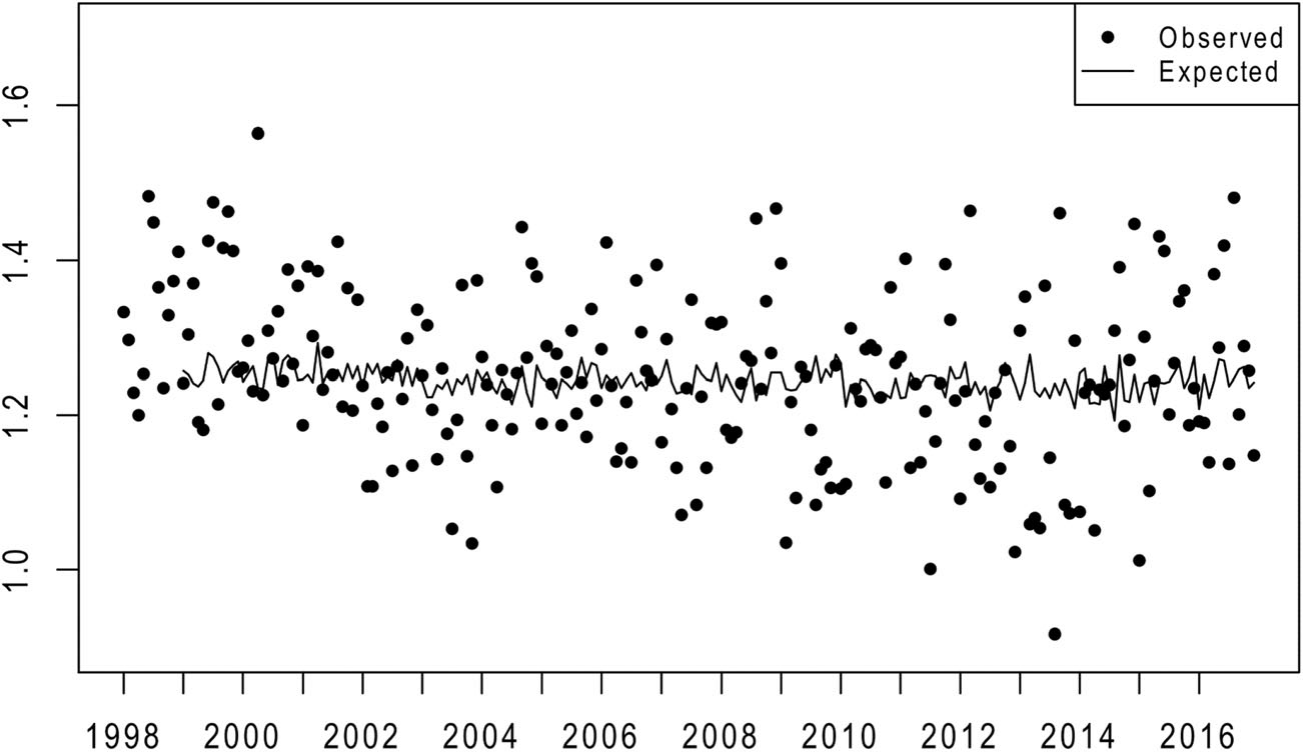
Observed (points) and expected (line) values of the natural logs of the monthly ratio of the conception cohort odds of periviable birth among African American to non-Hispanic white males (1/1998 to 12/2016)

**Fig. 2:**
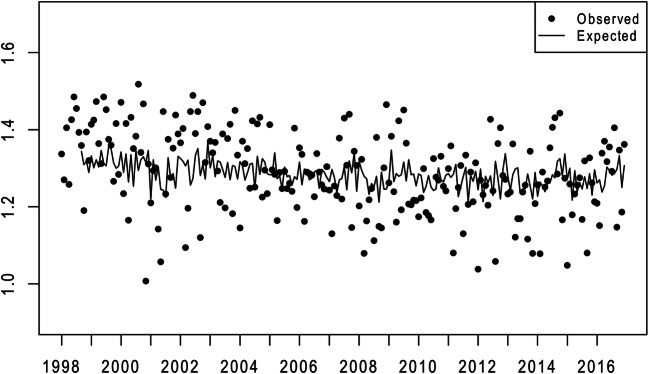
Observed (points) and expected (line) values of the natural logs of the monthly ratio of the conception cohort odds of periviable birth among African American to non-Hispanic white females (1/1998 to 12/2016)

Results from steps 1 and 2 appear graphically in Figs. 1 and 2. The points in these figures show the logged odds ratio of male and female periviable births yielded by conception cohorts conceived by African American and non-Hispanic white mothers in the 228 months beginning January 1998 and ending December 2016. The lines show the statistically expected (i.e., from autocorrelation) values of these ratios.

**Fig. 3:**
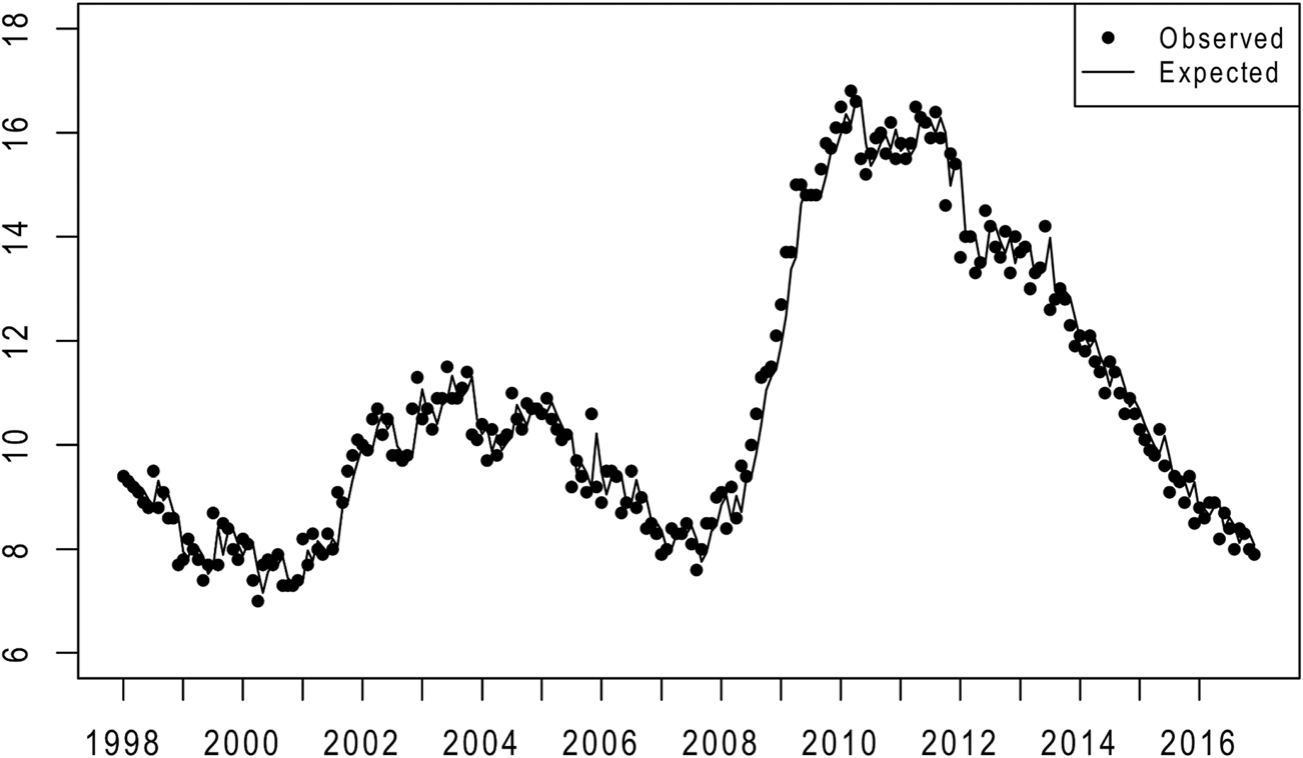
Observed (points) and expected (line) values of the seasonally adjusted monthly African American unemployment rate (1/1998 to 12/2016)

Figure 3 shows the expected (i.e., from autocorrelation), estimated in step 3, and observed values of the seasonally adjusted US African American unemployment rate for the test period. The difference between the observed and expected African American unemployment rates serves as our independent variable and shows the influence of the Great Recession in late 2008 and in 2009.

Table 1 shows the Box–Jenkins models that yielded the lines, or expected values, in Figs. 1, 2 and 3. The series for both males and females exhibited seasonality, although not identically, in that autoregressive parameters at 6 and 12 months appear in the models. The seasonal adjustment performed by the US Department of Labor precluded finding seasonality in the African American unemployment rate, but the Box–Jenkins method detected and adjusted trends in the series, as well as a moving average.

**Table 1:** Estimated coefficients (standard errors in parentheses) of the Box–Jenkins univariate models for the logged, sex-specific odds ratios of African American and non-Hispanic white periviable births and for the seasonally adjusted African American unemployment rate (*n*=228 monthly cohorts starting January 1998 and ending December 2016)

	Males	Females	African American unemployment rate
Differencing	None	None	At t-1
Constant	1.2435* (0.0087)	1.2834* (0.0106	None
Autoregressive parameters	At t-12 = 0.1544* (0.0669)	At t-2 = 0.2629* (0.0650) At t-6 = 0.1458* (0.0663)	None
Moving average parameters	None	None	At t-1 = 0.02703* (0.0624)

*P < 0.01; 2-tailed test

The estimated coefficients for our test model, estimated in step 4, appear in Table 2. Consistent with our expectations of a statistically detectable association for males past the first trimester of gestation, the coefficient (i.e., 0.0435) for unemployment 4 months after the conception of male cohorts appears detectably greater than 0. As implied by its standard error (0.0146) a coefficient of this size would appear by chance fewer than 5 times in 1000 experiments (single-tailed test). This finding implies that male conception cohorts yielded greater than expected differences between African American and non-Hispanic white odds of periviable birth when exposed to higher than expected African American unemployment in the 5th month of gestation. Also consistent with our expectations, this association appears greater than any of those between unemployment and the racial disparity for female neonates.

**Table 2:** Estimated coefficients (standard errors in parentheses) for equation predicting the logged, sex-specific odds ratios of African American and non-Hispanic white periviable births from differences between expected and observed values of the African American unemployment rate (*n*=228 monthly cohorts starting 1/1998 and ending 12/2016)

	Male	Female
Constant	1.2412** (0.0089)	1.2832** (0.0112)
Unemployment during:		
month of conception	0.0195 (0.0146)	0.0095 (0.0140)
1 month after conception	−0.0204 (0.0146)	0.0087 (0.0140)
2 months after conception	−0.0056 (0.0146)	−0.0103 (0.0145)
3 months after conception	0.0226 (0.0146)	0.0189 (0.0149)
4 months after conception	0.0435** (0.0146)	−0.0007 (0.0145)
5 months after conception	−0.0029 (0.0145)	0.0050 (0.0142)
6 months after conception	0.0141 (0.0146)	0.0131 (0.0141)
Autoregressive parameter(s)	at 12 = 0.1633* (0.0707)	at 2 = 0.2535** (0.0682) at 6 =0.1667* (0.0704)

*P < 0.01, 1-tailed test

**P < 0.005, 1-tailed test

The antilog of the 0.0435 coefficient shown in Table 1 suggests that the racial disparity for males increased by 4.45% for each percentage point the African American unemployment rate increased above expected. The greatest difference between observed and expected unemployment (i.e., 1.402) appeared in July 2005 implying that the cohort conceived in March yielded a 6.24% greater than expected racial disparity among males (i.e., 1.402 X 4.45). The median of the higher than expected differences between observed and expected African American unemployment was 0.359% implying that half the 105 exposed male cohorts exhibited racial disparities at least 1.6% greater than expected.

**Fig. 4:**
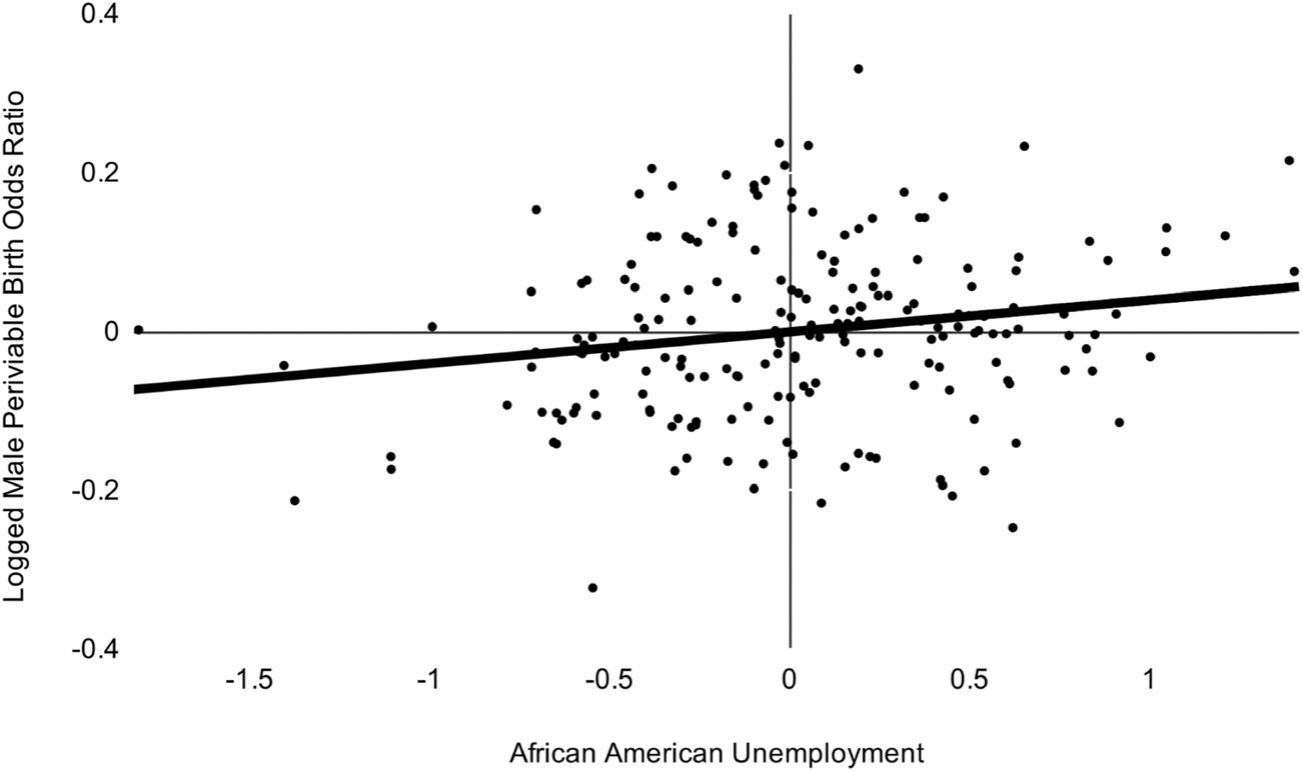
Scatter plot of the Box–Jenkins residuals of the natural logs of the monthly ratio of the conception cohort odds of periviable birth among African American to non-Hispanic white males over the Box–Jenkins residuals of the African American unemployment rate 4 months before cohort conception (1/1998 to 12/2016)

Figure 4 shows our principal finding as a scatter plot with best-fitting line. The Y axis shows the residuals of the Box–Jenkins model estimated for males in Step 2 (i.e., differences between the observed and expected values of the logged monthly ratio of conception cohort odds of periviable birth among African American to non-Hispanic white males). The X axis shows the residuals of the Box–Jenkins model estimated in Step 3 (i.e., the differences between expected and observed values of the African American unemployment rate) 4 months after the conception of the cohorts.

We conducted several additional analyses to gauge the robustness of our finding. First, we converted our dependent variable to the difference between, rather than ratio of, the odds of male periviable births for African Americans and non-Hispanic whites thereby invoking the logic of “difference-in-differences” tests common outside epidemiology. We applied steps 2 through 4 above to the differences and found the same result in that cohorts in the 5th month of gestation produced detectably greater disparities than expected when exposed to unexpectedly high African American unemployment.

Second, we applied steps 2 through 4 above to the odds of periviable birth for African American males alone to determine if our main finding reflected an increased risk among them rather than a decrease among non-Hispanic white males. Again, we found that pregnancies in the 5th month or gestation yielded more than expected periviable births when African American unemployment rose above expected values.

Third, we removed variables without detectable contributions to explained variance from our first test model for males and estimated it again. The coefficient for cohorts in the 5th month of gestation remained detectably greater than 0.

Last, we applied the methods of Chang, Tiao, and Chen [52] to our first model to determine whether outliers in the dependent variable had inflated the confidence interval of the residuals and led to false acceptance of the null for deleted variables. One outlier (i.e., April 2000) appeared but adjusting step 4 estimations for its effect did not change the results of our test.

## Discussion

Our findings support the argument that selection *in utero* induced by unemployment contributes to the disparity in male periviable births between African American and non-Hispanic white women. We discovered a greater than expected racial disparity in male periviable births among cohorts exposed to unexpectedly high unemployment among African Americans during the 5th month of gestation.

We focused on male infants because they predominate among periviable births and because theory suggests that risk of very early birth among male fetuses should respond more to the maternal stress response than should the risk among females. This male-specific response has implications for literature beyond that concerned with periviable birth. It reinforces, for example, the suspicion that the comparatively low and stable ratio of male to female births to African Americans over the preterm period arises, in part, from earlier stress-induced selection against male fetuses [4].

We used unemployment as a population stressor for the reasons noted above. Further research should estimate the dose response for other population stressors, including, for example, natural disasters, community violence, and racist political rhetoric, associated with preterm birth and suffered differently by racial or ethnic groups [53].

The welcomed rarity of periviable births led us to aggregate monthly conception cohorts across the entire US. We, however, acknowledge that the economic experience of African Americans likely varies substantially by place. Although the US Bureau of Labor Statistics does not publish time-series of race-specific unemployment at the subnational level, we encourage further research to identify times and places in which economic contraction plausibly induced unexpectedly high levels of selection *in utero* and periviable births to African American women.

African American births, like those among non-Hispanic whites, declined during the Great Recession [54]. The literature typically attributes these declines to postponed childbearing [55]. Consistent with this argument, unplanned births among underrepresented minorities and low socioeconomic groups also declined during the recession [56]. Other literature, however, suggests that some fraction of the decline may arise from selection *in utero* [57]*.*

Much of the literature concerned with preterm birth invokes the argument that the maternal stress response “dysregulates” the “pregnancy clock” that would otherwise have yielded a term infant [20, 21]. Prevention efforts based on this argument have, however, proved largely ineffective [58–60]. Ethicists have, moreover, noted that the dysregulation narrative reinforces a “disease” perspective that stigmatizes women who deliver preterm infants [61]. We argue instead that a portion of periviable births arise from increasingly effective clinical intervention into selection *in utero*—a well-regulated, adaptive mechanism conserved in healthy women of all races. Our argument suggests, consistent with our findings, that some portion of racial disparities in periviable births results from an adaptive biological response to a similar disparity in exogenous stressors encountered by African American and non-Hispanic white women. Our argument further suggests that prevention efforts should focus on more salutary regulation not only of gestation, but also of the environments in which African Americans reside.

## Conclusions

Public and private policies affect the distribution not only of stressors (e.g., workplace and residential toxins) but also of the resources (e.g., income transfers, lending practices) used to cope with stress. These policies, informed by cultural and social values, favor racial/ethnic groups differently and thereby create a “racialized” structure of risk for illness and disease [30, 62]. Public policy and private practices affecting employment opportunities have, for example, historically favored non-Hispanic whites over African Americans [30, 62]. The persistently higher unemployment rate among African Americans has, we believe, contributed to persistently higher rates of periviable birth. If so, then employment policies would seem a reasonable target for salutary interventions [63, 64].

## Data Availability

From publicly available vital statistics.

## Code Availability

From the corresponding author.
